# Thrombocytopenia: is it a prognostic factor for development of post-hemorrhagic hydrocephalus in neonates?

**DOI:** 10.1007/s00381-020-04790-5

**Published:** 2020-07-13

**Authors:** Ahmed El Damaty, Luca Giannoni, Andreas Unterberg, Heidi Baechli

**Affiliations:** Department of Neurosurgery, Heidelberg University Hospital, Heidelberg, Germany

**Keywords:** Germinal matrix, Hydrocephalus communicans, Intraventricular hemorrhage

## Abstract

**Purpose:**

Post-hemorrhagic hydrocephalus (PHH) is a rare but serious complication among premature babies in the neonatal intensive care unit. The causes of PHH are still not entirely understood, and its prevention and treatment are controversial. We tried to analyze the risk factors for such complication in our cohort.

**Methods:**

We reviewed our neonatology data bank and included all preterms below 28 weeks who were born in the period between 1999 and 2014 and suffered from an intraventricular hemorrhage (IVH). We reviewed gestational age, gender, birth weight, type of birth, IVH degree, comorbidities, therapy, complications, time to event, protein content of cerebrospinal fluid, and clinical follow-up.

**Results:**

We identified 180 patients, divided into two subgroups, “B1” with 37 cases (IVH + PHH) and “B2” with 143 cases (IVH − PHH). In group B1, the presence of IVH grades I, II, III, or IV was in 11%, 19%, and 70% respectively. Nineteen patients were treated with a ventricular access device (VAD) or external ventricular drain (EVD). A total of 20 shunts were implanted, with 11 revisions (55%). One patient suffered from thrombocytopenia. In subgroup B2, 51% showed IVH grade I, whereas severe IVH grades were only present in 22%. 25.9% suffered from thrombocytopenia. Thrombocytopenia was significantly higher in patients who did not develop PHH (*p* value: 0.002).

**Conclusion:**

According to our results, thrombocytopenia could play a decisive role in avoiding development of PHH as a sequel of IVH. We recommend a randomized controlled trial to assess the possible efficacy of antiplatelet drugs in avoiding PHH in this vulnerable group.

## Introduction

Despite the enormous development of treatment options and an increase in prenatal diagnostic procedures, intraventricular hemorrhage (IVH) remains one of the most common complications in premature births [1]. Post-hemorrhagic hydrocephalus (PHH) can develop after IVH due to obliteration of the arachnoid villi by microthrombi with subsequent inflammation and gliosis causing cerebrospinal fluid (CSF) outflow obstruction [2]. The incidence of IVH has decreased steadily in the last years [3–6]. However, the risk of developing IVH increases due to steadily improved medical care and intensive therapy; these advancements enabled premature babies to survive with a gestational age below the 28th week of pregnancy [7].

Premature babies are at increased risk of numerous comorbidities [8]. The general definition of prematurity includes all births before the end of the 37th week of gestation [9]. Preterms below the 28th week of pregnancy are called “extremely preterm” [10]. This particular age group develops intraventricular bleeding with a probability of 15–20% [11]. Although a downward trend in the incidence of IVH has been described in recent decades [12], the incidence of PHH requiring treatment appears to remain constant and is described with a probability of 25–50% [13]. Despite this fact, unfortunately, no clear guidelines for the treatment of PHH until now have been described, even an optimal time for performing surgical measures is still debatable, and despite adequate therapeutic measures, the occurrence of neurological sequelae still occur [1, 12].

The aim of this work is to identify the risk factors for the development of PHH in extreme preterms with IVH, as well as the evaluation of the therapies.

## Patients and methods

The study was approved by the local ethics committee. According to this approval, patient consent was not necessary because of the retrospective nature of the study. We did a retrospective review of our neonatology data bank and included all patients who were born in the period from Jan 1999 to Dec 2014 with a gestational age below 28 weeks and suffered from an IVH of any degree. We excluded from the study group all preterm infants with a gestational age above 28 weeks of gestation and those who were born before the end of the 28th week of gestation but had no IVH. Furthermore, all patients with a follow-up period of less than 1 year were excluded from the study. The following data was collected: date of birth; gestational age; gender; birth weight; type of birth; IVH degree (see Table 1); number of multiple pregnancies (single, twin, triplet); comorbidities (such as respiratory and cardiovascular diseases, thrombocytopenia, and sepsis); therapeutic measures such as external ventricular drain (EVD), ventricular access device (VAD), or ventriculoperitoneal (VP) shunt implantation, complications of treatment, period from first diagnosis of PHH to implantation of a VP shunt (time to event), protein content in CSF before shunt implantation, and follow-up (need for speech therapy, physiotherapy, developmental milestones, etc.). In our patients, we did not do neither lumbar punctures nor ventricular taps due to their possible high risk of injury and/or infection. We restricted our operative temporizing measures only to insertion of VAD or rarely EVD.

**Table 1:** Classification of intracranial hemorrhage in premature infants [14]

IVH grade	Description
Grade I	Bleeding is subependymal
Grade II	Bleeding is intraventricular without ventricular dilation
Grade III	Bleeding is intraventricular with ventricular dilation
Grade IV	Bleeding is intraventricular and intraparenchymal

In our study, we defined thrombocytopenia when the platelet count is below 150/nl; we differentiated between initial thrombocytopenia which was detected in the first blood sample coinciding with the development of intraventricular hemorrhage and transitional or secondary one which appeared with diagnoses associated with abnormal platelet counts, for example pregnancy-induced hypertension, infection, or necrotizing enterocolitis. Platelet transfusion consisted of 10 ml/kg (neonate’s weight at the time of transfusion) of unmodified platelet concentrate and resulted in an average increment of approximately 100/nl. This was performed whenever the platelet count falls below 100/nl, and signs of bleeding tendency or high risk of bleeding were noticed or in case of preoperative preparation.

All patients of the study, as they were extreme premature, received hydrocortison, phenobarbitone, vitamin K, surfactant, antibiotics, and inotropes whenever needed and were intubated and artificially ventilated until the lungs mature, and they could be weaned of the ventilator.

We divided our cohort into 2 groups: study group B including preterm infants who developed IVH and a matched control group K. Group B was subdivided into 2 subgroups: B1 including patients who suffered from IVH and developed PHH and B2 who suffered from IVH but did not develop PHH. All data were encrypted in self-created database in EXCEL and evaluated with the statistical programs SPSS (version 24) and EXCEL (office version 2010). The relationship between the large study group “B” and the control group “K” was considered. The control group consisted of patients who were born during the same period with a gestational age under the 28th week of pregnancy but had no IVH. The descriptive analysis was created using the mean, standard deviation, and frequency distributions of various parameters. Different tests were used to check for any existing significance between the two main or the two subgroups. The *t* test of independent samples was used for all continuous data. The Pearson chi-square test was performed for all nominal and ordinal data. The significance level was *p* < 0.05 for all tests.

## Results

**Fig. 1:**
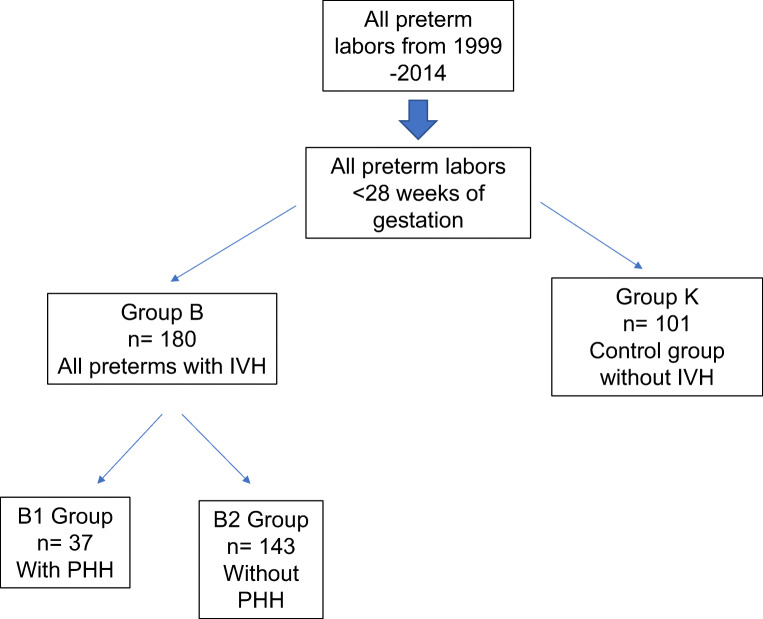
Patients’ flow chart

The study group “B” included 180 patients and the control group “K” included 101 patients. Furthermore, the study subgroup “B1” included 37 cases (patients with IVH and development of a PHH requiring therapy) and subgroup “B2” included 143 cases (patients with IVH, but without development of PHH); see Fig. 1. For both subgroups, there was a follow-up interval from a minimum of 1 to 15 years.

### Patients’ epidemiology

**Fig. 2:**
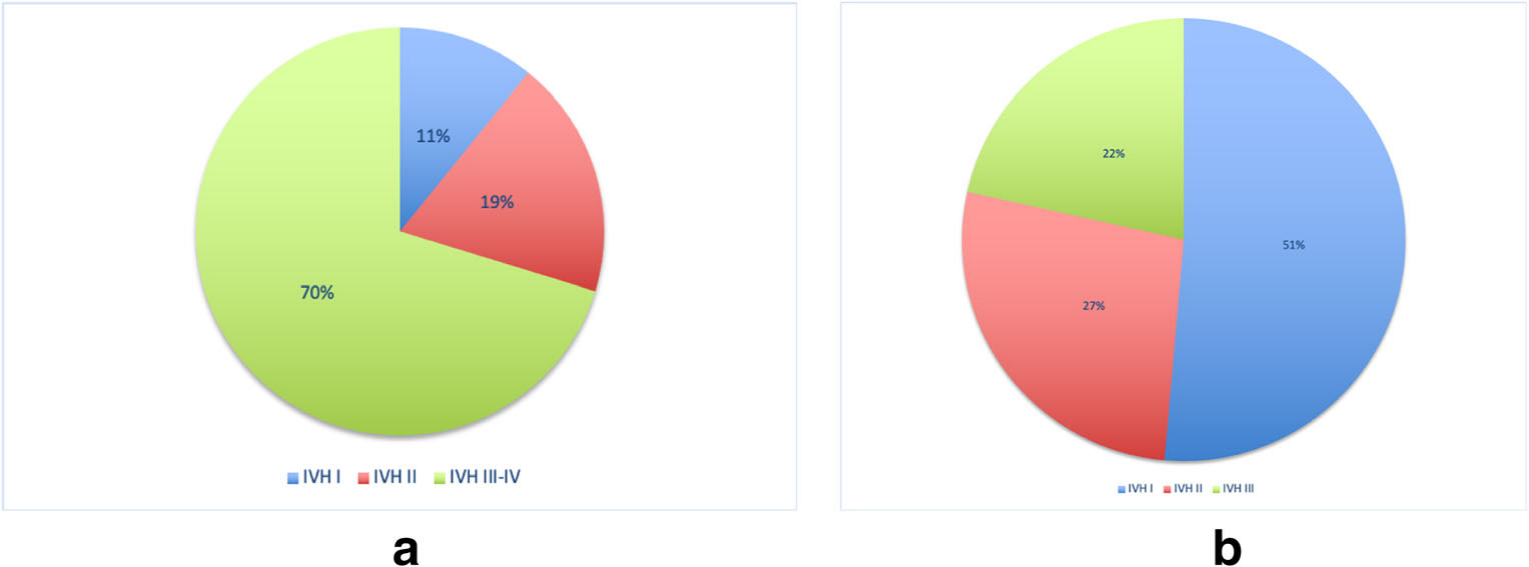
**a** Group B1 pie chart. **b** Group B2 pie chart

Study subgroup B1 (IVH + PHH) which comprised 37 patients showed an average gestational age of 25 weeks + 3 days (youngest 22 weeks + 6 days and the most mature 27 weeks + 6 days). The average birth weight was 0.785 kg, with a lowest birth weight of just 0.41 kg and the heaviest of 1.22 kg. Eleven percent of patients in subgroup B1 had an IVH grade I, 19% IVH grade II, and a clear majority with 70% IVH grades III or IV; see Fig. 2. While the gender ratio in bleeding grades I and II seemed to be balanced, intraventricular hemorrhage III–IV grades showed significantly a male predominance of 69%. Nineteen patients were treated with a VAD or EVD, and a total of 20 shunts were implanted, with 11 revisions (55%). Unfortunately, 10 patients (27%) died. There were 12 (32.4%) multiple pregnancies; see Table 2 for details.

**Table 2:** Group B1 findings

	Age	Birth weight (kg)	C-Section	Male (%)	Female (%)	VAD/EVD	Shunt	Revision	Mortality
IVH I	25 + 2	0.836	3	50	50	1	1	0	2 (50%)
IVH II	26 + 3	0.941	5	57	43	4	3	3	0 (0%)
IVH III–IV	25 + 3	0.735	16	69	31	14	16	8	8 (31%)
Total	25 + 3	0.785	24	24	13	19	20	11	10 (27%)

Subgroup B2 consisted of a total of 143 premature babies, all with IVH, but without PHH. The average age was also 25 weeks + 3 days (youngest 24 weeks + 5 days and the oldest 27 weeks + 6 days). The average weight was 0.748 kg. The majority of patients showed 51% grade I bleeding, whereas severe IVH grade III was only represented with 22%; see Fig. 2. The gender ratio of subgroup B2 was relatively balanced with 55.9% male and 44.1% female children. Of the 143 patients, 31 (22%) died. Twenty-eight percent of the cases were multiple pregnancies.

The control group K included 101 patients, and they were matched according to gestational age and birth weight. They showed similar averages in these categories as the examination group B. The gender ratio seems very balanced with 55.4% male and 44.6% female premature babies. There were almost one quarter of multiple pregnancies, and 10.9% of pre-treatment controls died.

### Comorbidities in subgroups

In subgroup B1, almost half (45.9%) of the patients had blood pressure fluctuations and almost all (91.9%) had respiratory or cardiovascular diseases. All premature infants with IVH grades I and II had demonstrable respiratory disease. In contrast, premature babies with an IVH grades III or IV only had 89%. In the aspect of cardiovascular diseases, the increase in the grade of bleeding denoted rather a decline in the occurrence of such coincidence, with only 54% of all patients with IVH grades III–IV having a cardiovascular disease. Twenty-two (59.5%) premature babies suffered from sepsis but only 1 patient in this group with IVH grade II initially from thrombocytopenia (2.7%).

**Fig. 3:**
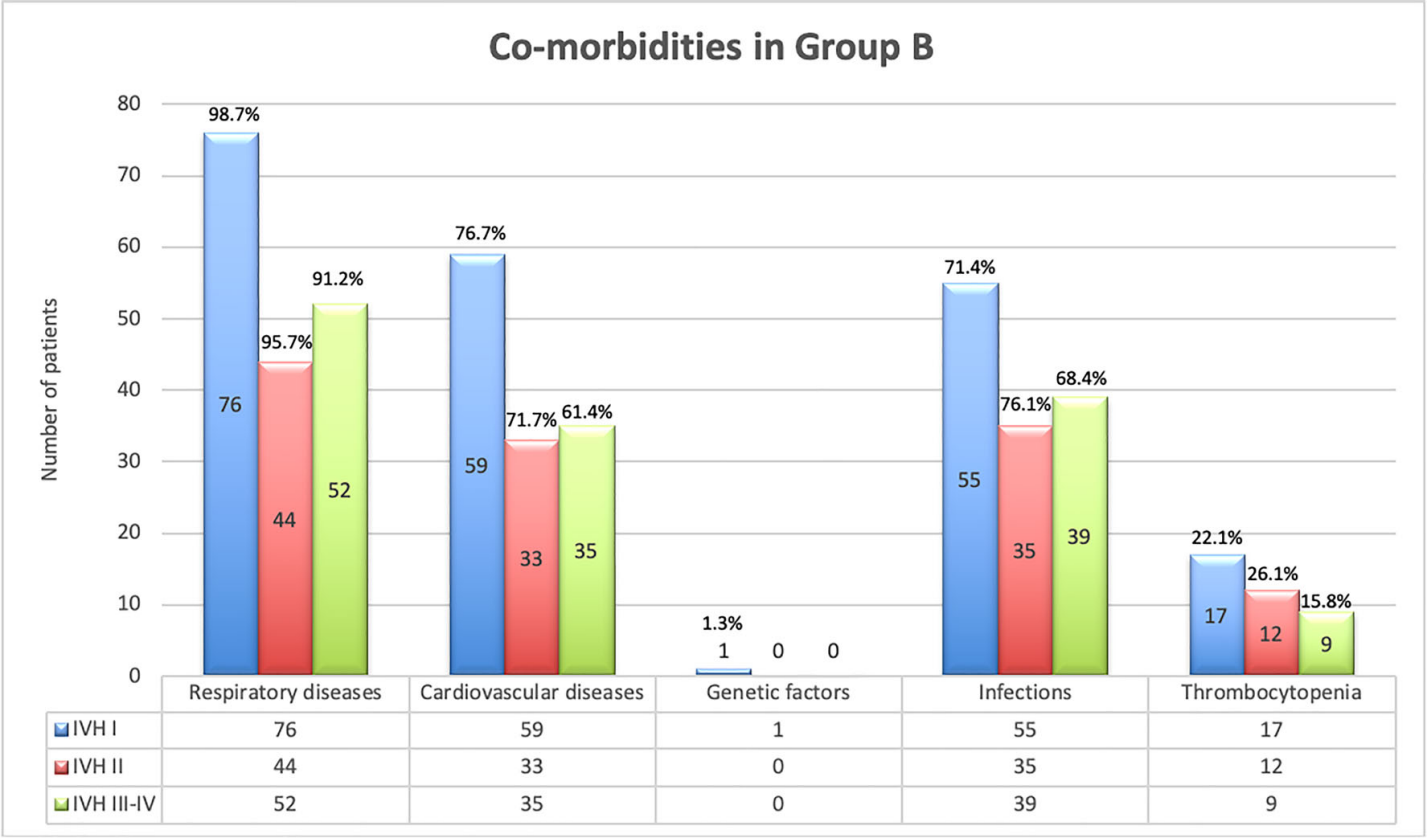
Comorbidities in group B

Subgroup B2 initially showed a similar trend in terms of comorbidities. Both the presence of respiratory and cardiovascular diseases decreases as the severity of intraventricular hemorrhage increases. 71.3% showed blood pressure fluctuations, and even 97.9% of the children had respiratory and cardiovascular diseases. Nearly three quarters (71.3%) of premature babies suffered from sepsis, and regarding the initial thrombocytopenia, we noticed a slight increase in incidence with higher IVH grades: in grade I, thrombocytopenia was in 23%, 28% in grade II, and 29% in grades III and IV, with a total percentage of 25.9%. When we collectively reviewed our study group B, we found most of the patients suffered from respiratory and/or cardiovascular problems which are very frequent in extreme preterms; see Fig. 3.

In the control group K, in 48.5%, they showed blood pressure fluctuations and all of them had a respiratory or cardiovascular disease without exception. In 68.3% of patients, they suffered from sepsis; only 11.9% had initial thrombocytopenia.

### Thrombocytopenia

First, the large study group B with a total of 180 patients was compared with the control group K with 101 patients. The presence of initial thrombocytopenia in the patients of study group B is higher in comparison with the “healthy” preterms of control group K; 21.1% versus 11.9% (*p* value 0.052); see Table 3. Additionally, we found in terms of blood pressure fluctuations statistical significance (*p* value of 0.004) and in mortalities (*p* value of 0.023). Here, it was shown that patients with IVH have significantly more blood pressure fluctuations and, above all, increased mortality.

**Table 3:** Statistical overview comparing results of groups B and K

	Group B (*n* = 180)	Group K (*n* = 101)	*p* value
Gestational age in days	178.2 ± 10.64	178.7 ± 10.39	0.685
Weight in kg	0.756 ± 0.209	0.751 ± 0.212	0.856
Gender (m/f)	m = 104 (57.8%) f = 76 (42.2%)	m = 56 (55.4%) f = 45 (44.6%)	0.705
Type of delivery (Cesarean section/normal)	CS = 137 (76.1%) Normal = 43 (23.9%)	CS = 78 (77.2%) Normal = 23 (22.8%)	0.832
IVH grade (I–IV)	I = 77 (42.8%) II = 46 (25.6%) III = 52 (28.9%) IV = 5 (2.8%)	/	n/a
Multiple pregnancy (yes/no)	y = 52 (28.9%) n = 128 (71.1%)	y = 25 (24.8%) n = 76 (75.2%)	0.456
Blood pressure variations (yes/no)	y = 119 (66.1%) n = 61 (33.9%)	y = 49 (48.5%) n = 52 (51.5%)	*0.004*
Respiratory-cardiovascular diseases (yes/no)	y = 174 (96.7%) n = 6 (3.3%)	y = 101 (100%) n = 0 (0%)	0.064
Blood transfusion (yes/no)	y = 15 (8.3%) n = 165 (91.7%)	y = 4 (4%) n = 97 (96%)	0.161
Sepsis/infection (yes/no)	y = 124 (68.9%) n = 56 (31.1%)	y = 69 (68.3%) n = 32 (31.7%)	0.921
Thrombocytopenia (yes/no)	y = 38 (21.1%) n = 142 (78.9%)	y = 12 (11.9%) n = 89 (88.1%)	0.052
Mortality (yes/no)	y = 39 (21.7%) n = 141 (78.3%)	y = 11 (10.9%) n = 90 (89.1%)	*0.023*

Blood pressure variations were significantly higher in patients with IVH in comparison to control group (*p* value 0.004). Also mortality was significantly higher in patients with IVH (*p* value 0.023)

In the second step, subgroup B1 which included 37 patients were compared with the subgroup B2 including 143 patients. We found that more premature babies of subgroup B2 exhibited initial thrombocytopenia and it was barely present in the patients of subgroup B1 (*p* value 0.002); see Table 4. Also, the IVH grade showed strong significance (*p* value < 0.00001) in terms of developing PHH requiring therapy. Regarding the blood pressure fluctuations, we observed that the premature babies of subgroup B2 had much more frequent blood pressure fluctuations (*p* value 0.004).

**Table 4:** Statistical overview comparing results of groups B1 and B2

	Group B1 (*n* = 37)	Group B2 (*n* = 143)	*p* value
Gestational age in days	180.4 ± 10.13	177.6 ± 10.72	0.153
Weight in kg	0.785 ± 0.205	0.748 ± 0.211	0.348
Gender (m/f)	m = 24 (64.9%) f = 13 (35.1%)	m = 80 (55.9%) f = 63 (44.1%)	0.327
Type of delivery (Cesarean section/normal)	CS = 24 (64.9%) Normal = 13 (35.1%)	CS = 113 (79%) Normal = 30 (21%)	0.072
IVH grade (I–IV)	I = 4 (10.8%) II = 7 (18.9%) III = 22 (59.5%) IV = 4 (10.8%)	I = 73 (51%) II = 39 (27.3%) III = 30 (21%) IV = 1 (0.7%)	< 0.0001
Multiple pregnancy (yes/no)	y = 12 (32.4%) n = 25 (67.6%)	y = 40 (28%) n = 103 (72%)	0.594
Blood pressure variations (yes/no)	y = 17 (45.9%) n = 20 (54.1%)	y = 102 (71.3%) n = 41 (28.7%)	*0.004*
Respiratory-cardiovascular diseases (yes/no)	y = 34 (91.9%) n = 3 (8.1%)	y = 140 (97.9%) n = 3 (2.1%)	0.069
Blood transfusion (yes/no)	y = 6 (16.2%) n = 31 (83.8%)	y = 9 (6.3%) n = 134 (93.7%)	0.052
Sepsis/infection (yes/no)	y = 22 (59.5%) n = 15 (40.5%)	y = 102 (71.3%) n = 41 (28.7%)	0.165
Shunt implantation (yes/no)	y = 20 (54.1%) n = 17 (45.9%)	/	n/a
Rickham reservoir (yes/no)	y = 19 (51.4%) n = 18 (48.6%)	/	n/a
Revisions (yes/no)	y = 17 (45.9%) n = 20 (54.1%)	/	n/a
Hydrocephalus (yes/no)	y = 37 (100%) n = 0 (0%)	/	n/a
Thrombocytopenia (yes/no)	y = 1 (2.7%) n = 36 (97.3%)	y = 37 (25.9%) n = 106 (74.1%)	*0.002*
Mortality (yes/no)	y = 10 (27%) n = 27 (73%)	y = 29 (20.3%) n = 114 (79.7%)	0.375

Thrombocytopenia was significantly higher in patients who developed IVH but did not proceed to PHH (*p* value 0.002)

## Discussion

### Management of PHH

The initial therapy of a diagnosed IVH aims to prevent further damage to the brain tissue through maintaining cerebral perfusion. The treatment of PHH in premature babies has been changed markedly in the last years [15]. A common consensus nowadays is to temporarily alleviate the pressure with an EVD or the implantation of a Rickham reservoir as a VAD until the necessity and feasibility of implantation of a VP shunt is decided [16]. Another study was devoted to neuroendoscopic lavage, which is proven to be helpful in newborns by breaking down the hematoma and washing out the residual bleeding; the initial evaluations showed a significantly lower shunt rate in the treated patients [17].

Lekic et al. [18] stated that early implantation of a VP shunt for the treatment of PHH is the method of choice. However, the VP shunt implantation can also bring many complications. Therefore, a non-invasive solution avoiding a shunt implantation would be desirable and could improve the quality of life of these patients [19]. A prospective multicenter study (TROPHY Register) deals specifically with the therapeutic measures of PHH and tries to establish clear guidelines and therapy recommendations for the treatment [20]. However, further clinical observation and evaluation are required.

### Grade of IVH, birth weight, and development of PHH

Previous studies showed that the more immaturity of a newborn, the higher the mortality rate [2, 12, 13]. Ten to fifteen percent of premature babies weighing less than 1500 g develop IVH and up to a third of them develop PHH [21]. The severity or extent of the germinal matrix bleeding correlates directly with the mortality of the premature babies; in premature infants with an IVH grade I, the death rate is only 10%, but with the severe grades III and IV, death rate is already at 26% and 47%, respectively [22]. Our study examined 180 patients, 37 of whom developed PHH representing around 20%. Twenty-six (70%) of these premature babies suffered from IVH grade III or IV. From the 143 patients who did not develop hydrocephalus, 73 patients (51%) suffered from IVH grade I. This clearly shows that the incidence of developing PHH increases drastically with the immaturity of the premature babies and especially the severity of the IVH.

### Correlation between thrombocytopenia and the development of PHH

Póvoa et al. [23] reported that fetal/neonatal alloimmune thrombocytopenia is one of the most common causes of severe thrombocytopenia in the newborn. It results from fetomaternal mismatch for human platelet alloantigens leading to antibody-mediated destruction of fetal platelets. In severe cases, intracranial hemorrhage may occur and lead to death or neurologic sequelae. On the contrary, Bu et al. [24] explained in adults the development of PHH as a sequel of IVH; they focused on the mechanisms of hydrocephalus after adult IVH, including blood clot blockage, barrier impairment, inflammation, and blood components. Hence, a normal coagulation function is needed to develop the full-blown picture of PHH. Clinically, hematoma volume after GMH-IVH is a prognostic indicator of future neurologic outcomes [25–27]. Various preclinical models of stroke have shown that rapid hematoma clearance after hemorrhagic stroke ameliorated inflammation and improved neurological deficits in the short and long terms [28]. Rappard et al. [29] found that the blood clot firmness is highly depending on the platelet count and that thrombocytopenia results in a soft unstable clot. Similarly, thrombocytopenia may help in the decrease of clot formation with its sequelae of blockage, barrier impairment, and inflammatory reaction, and even if the intraventricular hematoma is formed it is not firm and usually resorbs faster.

In our cohort, we found that initial thrombocytopenia was detected in more patients who developed IVH (group B) in comparison with the control group (group K) 21.1% vs 11.9% (*p* value 0.052) but statistically not significant. This could be explained either that thrombocytopenia is the cause for IVH through a bleeding tendency or as a sequel through consumption of platelets in forming the blood clot in the ventricular system. Regarding the two groups who developed IVH, we found a marked increase in the incidence of thrombocytopenia in the group who did not develop PHH (group B2) in comparison with those who developed PHH (group B1) 25.9% vs 2.7% (*p* value 0.002). This raises the assumption that the platelet count may be crucial in deciding whether, after development of the IVH, the cascade will continue to develop PHH or the process will be less severe with less damage and less sequelae evading the development of PHH.

TGF-β stimulates mesenchymal stem cells and fibroblasts, which produce ECM matrix proteins and deposit connective tissue (Bowen et al. 2013). TGF-β can be secreted from activated microglia, and TGF-β secretion can be induced by thrombin (Schuliga 2015). ECM production induced by TGF-β stimulation may deposit in the cerebroventricular system, disrupting CSF dynamics (Tada, Kanaji, & Kobayashi, 1994). A rabbit pup GMH model indicated TGF-β, fibronectin, and laminin expression levels were significantly increased in the ependymal and subependyma tissues after GMH (Cherian, Thoresen, Silver, Whitelaw, & Love, 2004a). Mice with transgenic TGF-β overexpression developed hydrocephalus with higher expression of ECM proteins in the brain than wild types (Wyss-Coray et al., 1995). In a clinical study, increased TGF-β1 and ECM protein expression in the CSF were associated with PHH development in preterm infants (Aquilina, Chakkarapani, & Thoresen, 2012; Douglas-Escobar & Weiss, 2012). The TGF-β1 isoform is mostly associated with PHH after IVH in neonates and adults (Gomes, Sousa Vde, & Romao, 2005). Intrathecal TGF-β1 injection in mice resulted in hydrocephalus development, and TGF-β1 expression was significantly increased in brains of neonatal rats with PHVD after intraventricular blood injection (Cherian, Thoresen et al., 2004a; Tada et al., 1994). Indeed, TGF-β1 was elevated in both animal models and premature infants with PHH, although some studies dispute this (Heep et al., 2004). In a rat GMH model, TGF-β1 was elevated within hours after GMH, but normalized by 24-h post-ictus (Tang, Chen et al., 2015). Additionally, inhibiting TGF-β1 ameliorated long-term PHH and neurocognitive deficits as well as reduced vitronectin and GFAP expression in rats (Manaenko et al. 2014). Although the mechanism of TGF-β signaling after GMH and its association with PHH development has been established, studies are lacking that discern the changes to CSF dynamics as a consequence of TGF-β signaling and fibrosis.

Transforming growth factor β1 (TGF-β1) is a platelet-derived cytokine involved in both normal wound healing and scarring [30]. TGF-β activates mesenchymal stem cells and fibroblasts, to produce extracellular matrix (ECM) proteins and deposit connective tissue [31]. TGF-β can be secreted from activated microglia, and its secretion can be induced by thrombin [32]. Monroe et al. [33] showed that platelets play a major role in localizing and controlling the burst of thrombin generation leading to fibrin clot formation. Additionally, the increased level of ECM proteins, induced by TGF-β stimulation, may lead to its deposition in the cerebroventricular system and disruption of the CSF dynamics causing hydrocephalus [19, 34]. Mice with transgenic TGF-β overexpression developed hydrocephalus with higher expression of ECM proteins in their brains than wild types [35]. Intrathecal TGF-β1 injection in mice resulted in development of hydrocephalus, and TGF-β1 expression was significantly higher in brains of neonatal rats with post-hemorrhagic ventricular dilatation after injection of blood intraventricular [34, 36]. In a clinical study, increased TGF-β1 and ECM protein expression in the CSF was associated with PHH development in preterm infants [37, 38]. Additionally, inhibiting TGF-β1 minimized the long-term PHH and neurocognitive deficits in germinal matrix hemorrhage (GMH) model in rats [39]. Although the mechanism of TGF-β signaling after GMH and its association with PHH development has been established, we are still in short of studies that detect the changes to CSF dynamics as a consequence of TGF-β signaling and fibrosis.

To summarize, platelets play a decisive role in fibrin clot formation and to a major extent decide its firmness and stability. Additionally, platelets through mediating TGF-β complex play an important role in the development of the astrogliosis disrupting the CSF dynamics intraparenchymally and contributing to the development of PHH. Hence, we hope that through further studies focusing on these information, we could help to minimize the incidence of development of PHH following IVH in neonates.

## Conclusion

According to our results, we found that the grade of intraventricular hemorrhage defines at utmost the incidence of development of post-hemorrhagic hydrocephalus. Also, we found that the incidence of shunt complications is in direct proportion to the initial grade of IVH; although in all patients, irrespective to the IVH grade, the VP shunt was inserted with almost similar protein content in CSF. Finally, we found that thrombocytopenia was significantly higher in patients who developed intraventricular hemorrhage but did not develop post-hemorrhagic hydrocephalus. Hence, thrombocytopenia could play a decisive role in avoiding development of PHH as a sequel of IVH. We recommend a randomized controlled trial to assess the possible efficacy of antiplatelet drugs in avoiding PHH in this vulnerable group.
